# Acceptance, Barriers, and Facilitators to Implementing Artificial Intelligence–Based Decision Support Systems in Emergency Departments: Quantitative and Qualitative Evaluation

**DOI:** 10.2196/36501

**Published:** 2022-06-13

**Authors:** Ryo Fujimori, Keibun Liu, Shoko Soeno, Hiromu Naraba, Kentaro Ogura, Konan Hara, Tomohiro Sonoo, Takayuki Ogura, Kensuke Nakamura, Tadahiro Goto

**Affiliations:** 1 Faculty of Medicine The University of Tokyo Tokyo Japan; 2 TXP Medical Co Ltd Tokyo Japan; 3 Critical Care Research Group The Prince Charles Hospital Brisbane Australia; 4 Department of Palliative Care Southern Tohoku General Hospital Fukushima Japan; 5 Department of Emergency and Critical Care Medicine Hitachi General Hospital Ibaraki Japan; 6 Department of Economics University of Arizona Tucson, AZ United States; 7 Department of Emergency and Critical Care Medicine Saiseikai Utsunomiya Hospital Tochigi Japan

**Keywords:** clinical decision support system, preimplementation, qualitative, mixed methods, artificial intelligence, emergency medicine, CDSS, computerized decision, computerized decision support system, AI, AI-based, CFIR, quantitative analysis

## Abstract

**Background:**

Despite the increasing availability of clinical decision support systems (CDSSs) and rising expectation for CDSSs based on artificial intelligence (AI), little is known about the acceptance of AI-based CDSS by physicians and its barriers and facilitators in emergency care settings.

**Objective:**

We aimed to evaluate the acceptance, barriers, and facilitators to implementing AI-based CDSSs in the emergency care setting through the opinions of physicians on our newly developed, real-time AI-based CDSS, which alerts ED physicians by predicting aortic dissection based on numeric and text information from medical charts, by using the Unified Theory of Acceptance and Use of Technology (UTAUT; for quantitative evaluation) and the Consolidated Framework for Implementation Research (CFIR; for qualitative evaluation) frameworks.

**Methods:**

This mixed methods study was performed from March to April 2021. Transitional year residents (n=6), emergency medicine residents (n=5), and emergency physicians (n=3) from two community, tertiary care hospitals in Japan were included. We first developed a real-time CDSS for predicting aortic dissection based on numeric and text information from medical charts (eg, chief complaints, medical history, vital signs) with natural language processing. This system was deployed on the internet, and the participants used the system with clinical vignettes of model cases. Participants were then involved in a mixed methods evaluation consisting of a UTAUT-based questionnaire with a 5-point Likert scale (quantitative) and a CFIR-based semistructured interview (qualitative). Cronbach α was calculated as a reliability estimate for UTAUT subconstructs. Interviews were sampled, transcribed, and analyzed using the MaxQDA software. The framework analysis approach was used during the study to determine the relevance of the CFIR constructs.

**Results:**

All 14 participants completed the questionnaires and interviews. Quantitative analysis revealed generally positive responses for user acceptance with all scores above the neutral score of 3.0. In addition, the mixed methods analysis identified two significant barriers (System Performance, Compatibility) and two major facilitators (Evidence Strength, Design Quality) for implementation of AI-based CDSSs in emergency care settings.

**Conclusions:**

Our mixed methods evaluation based on theoretically grounded frameworks revealed the acceptance, barriers, and facilitators of implementation of AI-based CDSS. Although the concern of system failure and overtrusting of the system could be barriers to implementation, the locality of the system and designing an intuitive user interface could likely facilitate the use of optimal AI-based CDSS. Alleviating and resolving these factors should be key to achieving good user acceptance of AI-based CDSS.

## Introduction

Clinical decision support systems (CDSSs) are computerized tools that are developed to assist clinicians in their decision-making processes with the ultimate goal of improving patient outcomes [1]. CDSSs support clinicians by employing various functions, including diagnostic support, disease management, prescription control, and drug control [1]. These CDSSs have been continuously developed over the past years and have become increasingly available in all areas of health care, including the emergency care setting [2-4]. In addition, the rapid development of computer science has led to advancements in artificial intelligence (AI)-based CDSSs [5], and the development of electronic health record (EHR) systems has enabled researchers to advance models and systems in the health care setting [6]. In the emergency department, physicians need to maximize their performance in a limited amount of time to deal with the high urgency and severity of the patients’ conditions. To address such needs of emergency physicians, multiple CDSSs, such as alert systems and diagnostic imaging support systems, have been developed [7,8].

Despite the increased availability of CDSSs, including AI-based CDSSs in emergency care settings, the use of these systems is limited and has yet to achieve widespread implementation [1,9]. Several studies, mostly those outside emergency care settings, have identified reasons for the low usage and/or effectiveness of CDSSs. For example, studies have attributed the lack of usability, lack of integration with host systems, lack of time to effectuate advice, and alert fatigue to the low usage of CDSSs [9-11]. In addition, as medical device approval is needed in Japan for most CDSSs, time and effort are needed for implementation. Despite such knowledge regarding the implementation of CDSSs, little is known about the acceptance, barriers, and facilitators of AI-based CDSSs in the emergency care setting.

To address this knowledge gap, we aimed to evaluate the acceptance, barriers, and facilitators to implementing AI-based CDSSs in the emergency care setting through the opinions of physicians on our newly developed, real-time AI-based CDSS, which alerts ED physicians by predicting aortic dissection based on numeric and text information from medical charts, by using the Unified Theory of Acceptance and Use of Technology (UTAUT; for quantitative evaluation) [12] and Consolidated Framework for Implementation Research (CFIR; for qualitative evaluation) frameworks [13].

## Methods

### Study Design, Setting, and Participants

This is a mixed methods (ie, quantitative and qualitative), cross-sectional study of 14 physicians from two community, tertiary care hospitals in Japan (6 transitional year residents with 0-2 years of clinical experience, 5 emergency medicine residents with 3-5 years of clinical experience, and 3 emergency physicians with 5 years of clinical experience). The qualitative sampling used convenience sampling. This study was performed from March to April 2021. The physicians participated in a 1.5-hour session consisting of the following three sections: (1) a brief introduction of the system and the focus of the research, (2) actual use of the AI-based CDSS deployed on the internet with clinical vignettes of model cases, and (3) participation in a mixed methods evaluation consisting of a UTAUT-based questionnaire with a 5-point Likert scale and a CFIR-based semistructured interview. All interviews were moderated by two of the four research team members (RF, TG, KL, SS).

**Figure 1:**
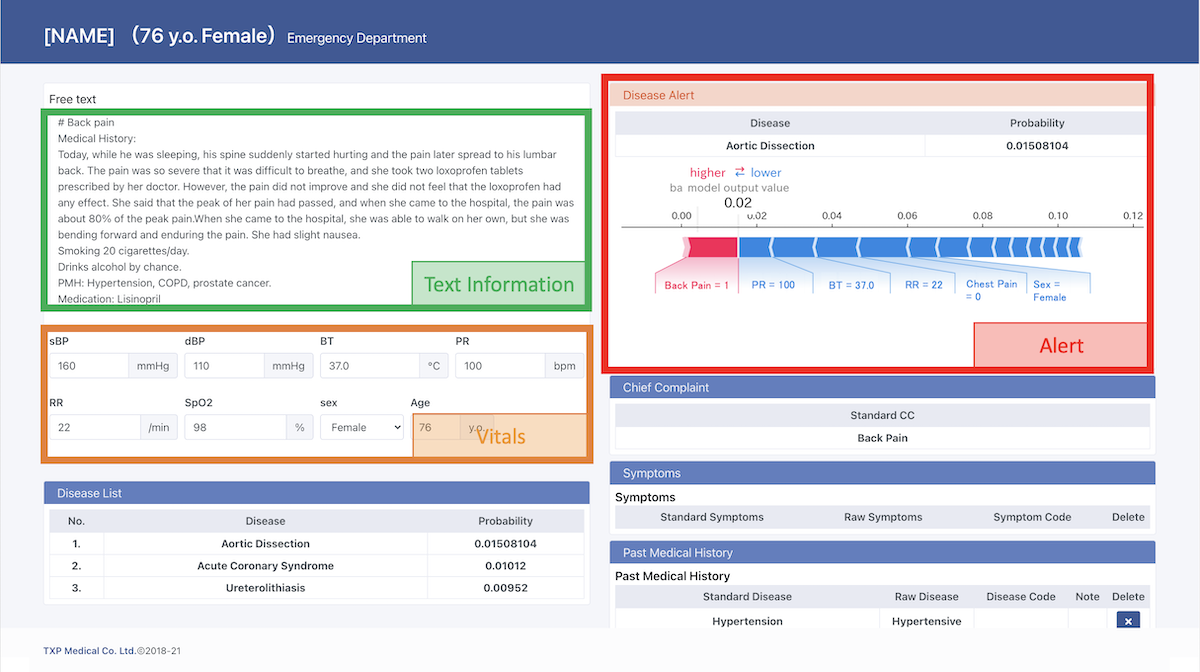
A real-time clinical decision support system with Emergency Alert System for predicting aortic dissection based on numeric and text information from medical charts (eg, chief complaints, medical history, vital signs) organized using natural language processing.

First, the moderator gave a brief introduction about the focus of the research and the CDSS, including how alerts are based on machine learning models. Verbal consent for participation in the research was obtained from each participant. In the second section, the participants used the AI-based CDSS deployed on the internet (Figure 1) with clinical vignettes of model cases created by two emergency physicians who authored this study (TG and KL). For this study, we implemented a machine learning–based CDSS for aortic dissection that consisted of an emergency alert system. We prepared both typical and atypical cases of the disease (Table S1 in Multimedia Appendix 1). Typical/atypical cases are defined by an author (RF) and confirmed by an emergency physician coauthor (TG). During the session, the moderator answered questions concerning the aim and background of the research, but questions on how to use the system and about the user interface were taken later in the interview to ensure that the explanations of the system were the same among participants. In the third section, the physicians participated in a mixed methods evaluation consisting of a UTAUT-based questionnaire with a 5-point Likert scale and a CFIR-based semistructured interview. All of these sessions were conducted using online video chat tools considering the infection risks of COVID-19.

The interviews were recorded using an audio recording device for ease of transcription and review. Data were transferred from the device following each interview and transcribed verbatim. The interview transcriptions were uploaded onto the MaxQDA 12 software (VERBI GmbH) for qualitative analysis [14]. All interviews were deidentified; a code was allocated to each interview, and personal identifiers were removed from the data. The codes were allocated based on the participant’s medical experience: transitional year residents (TYR1 to TYR6), emergency medicine residents (EMR1 to EMR5), and emergency physicians (EP1 to EP3).

### Proposed AI-Based CDSS

By using data from 27,550 emergency department patients from a tertiary care hospital in Japan, we first developed a real-time CDSS consisting of the Emergency Alert System, which notifies emergency department physicians by predicting aortic dissection based on numeric and text information from medical charts (eg, chief complaints, medical history, vital signs) organized using natural language processing (Figure 1). Aortic dissection was chosen as a pilot disease since it is an emergent, potentially fatal disease with numerous symptoms that can mimic non–life-threatening conditions [15]. The Emergency Alert System predicts the probability of the disease using real-time data input on the computer screen. The model used for prediction was developed using the XGBoost model [16] and hyperparameters were determined by 5-fold cross-validation to maximize the area under the receiver operator characteristic curve (AUROC). The AUROC of the model was 0.901 (95% CI 0.840-0.962). In an attempt to address anchoring bias while avoiding alert fatigue, the Emergency Alert System displays an alert when the probability of aortic dissection changes sharply (greater than 4 times the baseline risk) or exceeds the prespecified threshold of a predicted probability of 9.0%. In addition, to enhance the interpretability of the alert, the contribution of each feature (eg, chief complaint or systolic blood pressure) was calculated and was added to the alert on the screen as Shapley Additive exPlanations values [17]. The degree to which each feature contributed both positively and negatively was shown as a bar chart. To fully conduct the session online, this system was deployed on the internet via Amazon Web Services.

### Theoretical Framework Selection for Quantitative and Qualitative Evaluation

We used the UTAUT model to quantitatively evaluate users’ willingness to accept the proposed AI-based CDSS. The UTAUT model is composed of six main constructs that impact technology adoption: (1) effort expectancy, (2) performance expectancy, (3) social influence, (4) facilitating environment, (5) attitude toward using technology, and (6) behavioral intention [12]. We chose quantitative assessment for UTAUT because its components have relationships that have been determined by previous studies. Because this model does not consider the unique characteristics of the clinical setting (eg, limited time and human resources in the emergency department setting) [18], we further adopted qualitative research techniques based on the CFIR to further identify barriers and facilitators of the AI-based CDSS [13].

The CFIR was chosen because it is a relatively new framework that synthesizes prior research evidence into one consolidated framework with multiple constructs. In addition, the CFIR has flexibility in assessing implementation barriers and facilitators of research findings and innovations [13]. The CFIR consists of 39 constructs organized into 5 major domains found to influence the successful implementation of innovative programs. The domains assess the following characteristics of innovative programs: (1) intervention characteristics, (2) outer setting, (3) inner setting, (4) characteristics of individuals, and (5) process [13].

### Quantitative Analysis for UTAUT Questionnaire

The questionnaire for quantitative analysis was developed to investigate user attitudes toward the CDSS. A total of 23 questions were included in the questionnaire; of these, 21 represented concepts from the UTAUT and 2 questions were added for basic characteristics (age and sex). The phrasing of the questions was based on the original UTAUT article [19].

The questionnaire consisted of questions about the constructs of UTAUT: performance expectancy, effort expectancy, social influence, facilitating conditions, attitude toward using technology, and behavioral intention. Two to six questions were created to assess each domain, and each question was answered with a 5-point Likert scale. To confirm whether the UTAUT subconstructs are reliable in measuring the same construct, Cronbach α was calculated as a reliability estimate [20].

### Qualitative Analysis for CFIR Questionnaires

To objectively report results, the qualitative analysis followed the Consolidated Criteria for Reporting Qualitative Research (COREQ) guidelines [21]. Interviews were conducted based on CFIR [22]. For this study, the CFIR domains aligned with the following entities: intervention characteristics (Emergency Alert System), outer setting (community, tertiary care hospitals), inner setting (emergency department), and characteristics of individuals (emergency clinicians who piloted the Emergency Alert System). The process domain, which describes how implementation should be enacted, was excluded because the system was in a preimplementation phase and it was thought to be irrelevant to the study. Overall, 16 constructs from 4 domains were selected for the semistructured interview (Table 1).

**Table 1:** Consolidated Framework for Implementation Research domains and constructs.

Domain				Constructs
**I. Intervention characteristics^a^**				
	A		Intervention Source	
	B^a^		Evidence Strength and Quality	
	C^a^		Relative Advantage	
	D^a^		Adaptability	
	E		Trialability	
	F		Complexity	
	G^a^		Design Quality and Packaging	
	H		Cost	
**II. Outer setting^a^**				
	A^a^		Patient Needs and Resources	
	B		Cosmopolitanism	
	C		Peer Pressure	
	D^a^		External Policy and Incentives	
**III. Inner setting^a^**				
	A		Structural Characteristics	
	B		Networks and Communications	
	C^a^		Culture	
	**D^a^**		Implementation Climate	
		1^a^	Tension for Change	
		2^a^	Compatibility	
		3^a^	Relative Priority	
		4^a^	Organizational Incentives and Rewards	
		5	Goals and Feedback	
		6	Learning Climate	
	**E^a^**		Readiness for Implementation	
		1	Leadership Engagement	
		2^a^	Available Resources	
		3^a^	Access to Knowledge and Information	
**IV. Characteristics of individuals^a^**				
	A^a^		Knowledge and Beliefs about the Intervention	
	B		Self-efficacy	
	C		Individual Stage of Change	
	D		Individual Identification with Organization	
	E		Other Personal Attributes	
**V. Process**				
	A		Planning	
	B		Engaging	
	**C**		Executing	
		1	Opinion Leaders	
		2	Formally Appointed Internal Implementation Leaders	
		3	Champions	
		4	External Change Agents	
	D		Reflecting and Evaluating	

^a^The domains and constructs selected for the semistructured interview in this study.

The framework analysis approach [23,24] was used during the study to determine the relevance of the CFIR constructs. The framework analysis followed the 5-step process outlined by Richie and Spencer [25]: (1) familiarization, (2) identifying a thematic framework, (3) indexing, (4) charting, and (5) mapping/interpretation. The analysis was an ongoing iterative process. We conducted multiple reviews of the transcripts and video data to become familiar with the data (Step 1) and identify initial themes that were reflexive and interactive (Step 2). Analyses were initiated as soon as the first interview was completed and were continued concurrently with data collection to help determine when new information was no longer being generated from interviews. The team used the CFIR as the a priori framework, and the codes identified during the familiarization process were added to the CFIR. The codes also reflected relevant CFIR constructs across the 5 domains and were indexed to sections of the transcripts (Step 3). Sections of the transcripts were charted into themes and a summary matrix was organized with CFIR domains and constructs (Step 4). Two analysts reviewed the codes and associated themes multiple times to check for potential bias, to ensure they reflected participants’ words and that their interpretation of the interviews was credible (Step 5).

### Ethical Approval

We have confirmed with the ethics committee of TXP Medical Co Ltd that this study can be waived from ethical approval as it did not involve any patients.

## Results

### Overview

All 14 participants completed the questionnaires and interviews. The participant demographics and characteristics are shown in Table 2. All participants reported that they were unfamiliar or very unfamiliar with information technology. The UTAUT-based questionnaire lasted 3-7 minutes and the CFIR-based semistructured interview lasted 40-60 minutes. An interview of one participant was rescheduled due to network problems.

**Table 2:** Participant demographics (N=14).

Demographic			Participants, n (%)
**Specialty**			
	Transitional year resident	6 (43)	
	Emergency medicine resident	5 (36)	
	Emergency physician	3 (21)	
**Gender**			
	Male	9 (64)	
	Female	5 (36)	
**Age group (years)**			
	20-29	7 (50)	
	30-39	6 (43)	
	40-49	1 (7)	
**Information technology familiarity**			
	Very familiar	0 (0)	
	Familiar	0 (0)	
	Neutral	0 (0)	
	Unfamiliar	3 (21)	
	Very unfamiliar	11 (79)	

### Quantitative Analysis

For the 6 UTAUT constructs investigated, Table 3 lists the mean and standard deviation of the Likert scale for each question and the Cronbach α reliability statistics for each concept. Although the Cronbach α values of Facilitating Conditions and Social Influence were less than .6, other constructs exhibited good reliability within the recommended range of Cronbach α>.60. The analysis revealed generally positive responses for user acceptance, with high scores on Attitude and Intention to Use (mean 3.43, SD 0.76). Although 79% (11/14) of the participants were not familiar with information technology, their perceived complexity of the system was low (mean 4.14, SD 0.72).

**Table 3:** Construct reliability and mean (SD) scores for the Unified Theory of Acceptance and Use of Technology–based questionnaires (5-point Likert scale).

Construct		Mean (SD)	Cronbach α
**Performance Expectancy (PE)**			.638
	PE1: I would find the system useful in my job.	4.07 (0.73)	
	PE2: Using the system enables me to accomplish tasks more quickly.	3.14 (0.66)	
	PE3: Using the system improves the quality of the work I do.	3.57 (0.76)	
	PE4: Using the system enhances my effectiveness on the job.	3.86 (0.66)	
	PE5: If I use the system...My coworkers will perceive me as competent.	2.86 (0.95)	
**Effort Expectancy (EE)**			.690
	EE1: My interaction with the system would be clear and understandable.	4.36 (0.63)	
	EE2: I would find the system to be flexible to interact with.	3.57 (0.65)	
	EE3: It would be easy for me to become skillful at using the system.	4.50 (0.52)	
	EE4: Working with the system is so complicated, it is difficult to understand what is going on.	3.36 (0.50)	
	EE5: Using the system involves too much time doing mechanical operations (eg, data input).	3.00 (0.96)	
	EE6: My interaction with the system is clear and understandable.	3.86 (1.10)	
**Social Influence (SI)**			.499
	SI1: People who influence my behavior think that I should use the system.	3.00 (0.96)	
	SI2: Having the system is a status in my organization.	3.64 (1.45)	
**Facilitating Conditions (FC)**			.564
	FC1: I have the knowledge necessary to use the system.	3.14 (1.03)	
	FC2: Given the resources it takes to use the system, it would be easy for me to use the system.	4.21 (1.05)	
	FC3: A specific person (or group) is available for assistance with system difficulties.	4.07 (0.83)	
**Attitude Toward Using Technology (AT)**			.760
	AT1: Using the system is a bad/good idea.	4.14 (0.77)	
	AT2: I have fun using the system.	3.36 (0.84)	
**Behavior Intention (BI)**			.740
	BI1: I prefer to work with the system.	3.36 (0.84)	
	BI2: I intend to use the system in the next 3 months.	3.43 (0.76)	

### Qualitative Analysis

#### Overview

Table 4 presents the primary codes and count data differentiated by the CFIR domain and construct, as well as whether codes were barriers to or facilitators of Emergency Alert System implementation and adoption. Only relevant CFIR domains and constructs were coded and presented here. There were four key factors in the implementation of the system: Evidence Strength and Quality, Relative Advantage, Design Quality and Packaging, and Compatibility. Other factors influencing the implementation can be found in Table S2 in Multimedia Appendix 1.

**Table 4:** Primary codes and count data differentiated by Consolidated Framework for Implementation Research domain and construct.

Constructs		Barriers	Count		Facilitators		Count	
**I. Intervention Characteristics**								
	**Evidence Strength and Quality**							
		Distrust of the results	1		Sample size was enough for developing the model		9	
					Local trends of disease		1	
	**Relative Advantage**							
		Unnecessary for experienced emergency physicians	1		Potential to reduce misdiagnoses		1	
		Unnecessary for typical cases	6		More useful than diagnostic rules		2	
		Alternatives to the system are enough	2		Never seen similar systems		8	
		Can bias physicians’ decision-making	2		Can aid diagnosis for difficult cases		6	
		Limited use cases	1		Good for information sharing		1	
					Useful for unexperienced physicians		3	
**III. Inner Settings**								
	**Design Quality and Packaging**							
		Unable to find when the system shows alerts	1		Easy to use and not interruptive		10	
		Potentially distracting for comorbidities	1		Summary board was informative		1	
					Real-time alerts were intuitive		2	
	**Compatibility**							
		Anxious if the system is not working properly	1		Easily integrated with existing workflow		14	
		Affects typing speed	1					
		Fear of system failure	1					

#### Evidence Strength and Quality

Nine key informants emphasized that the sample size (>10,000) was enough for developing models. Indeed, some practitioners noted that systems based on data from a single or a few hospitals can be more beneficial because they would incorporate local trends of disease.

The sample size for the development of the model is not a problem for me. There are many more diagnostic rules that have less evidence than Emergency Alert System.

EMR1

I don’t think the evidence should be considered weak just because the models are developed based on local data. Rather, I think it is beneficial because it could incorporate and reflect local trends of disease presentation.

EP3

#### Relative Advantage

Two informants recommended the system as a good alternative to diagnostic charts, in that it can reduce misdiagnoses. They implied Emergency Alert System showed superiority in that recollection is easier and it can help in pinpointing differential diagnoses, especially in atypical cases. Nevertheless, informants stated that the system would not be useful for typical cases in that the differential diagnoses would not change with the alert. Three informants added that the system can trigger a differential diagnosis for inexperienced physicians, and two informants argued that it may bias the clinician’s decisions.

It is less likely to leave out diseases for experienced physicians. It is not necessary especially for physicians who have experience in the emergency department.

EP2

Easier to think of the differential diagnoses than a diagnostic chart.

TYR3

I think it would not be helpful for typical cases, though it can definitely be an aid for cases that are difficult to make the diagnosis.

TYR3

I feel that being alerted to a certain disease can cause bias, and it may lead to misdiagnoses.

EP2

#### Design Quality and Packaging

Ten informants talked about the design quality, and all of them considered the design to be neither interruptive for daily practice nor effortful and found it useful for medical practice. Moreover, the summary board made by the natural language processing algorithm was noted to be well visualized as a summary of information. Two informants mentioned the effect of real-time prediction and that the alert was intuitively visualized. However, one informant mentioned that if the system was extended to other diseases, the additional information could be complex and interruptive.

It is easy to see changes in the probability of the target disease, but I did not understand the timing of the alert.

TYR6

The summary screen was easy to understand.

EMR2

It is good that the alert comes out in real time.

TYR2

The system is simple, but I am concerned that alerts with many diseases would make it complex and interruptive.

EMR3

#### Compatibility

All informants noted that the system can be integrated with the existing workflow processes and practices. However, one informant stated that the system affected typing speed and could interfere with the clinical workflow if it were to stop working or freeze.

It is useful to obtain additional information without extra effort.

EMR3

Medical care can be done as usual. No difference from a regular electronic medical record.

TYR5

The system affects typing speed a bit. My biggest concern is that the system may interfere with the flow if the system freezes, or even worse, stops.

EP1

## Discussion

### Principal Findings

This study employed a mixed methods approach to analyze barriers to and acceptance of the implementation of AI-based CDSS. The quantitative analysis revealed generally positive responses for user acceptance and the qualitative analysis identified two significant barriers (System Performance, Compatibility) and two major facilitators (Evidence Strength, Design Quality) to implementation of AI-based CDSS. To our knowledge, this is the first study to analyze barriers, facilitators, and acceptance of AI-based CDSS implementation in an emergency care setting using a mixed methods approach.

### User Acceptance

The quantitative analysis showed positive scores for Attitude and Intention to Use. Employing the UTAUT model, the achieved mean score of 3.63 indicated that if the Emergency Alert System were to be developed from the current prototype into a full software product, it would likely be well accepted by its users, as all scores were above the neutral score of 3, indicating favorable attitudes toward the use of the system. Nonetheless, since misdiagnoses could be life-threatening in the emergency setting, the barriers and facilitators identified in the qualitative analysis should be addressed thoroughly for the system to be well accepted.

### Barriers

#### System Performance

The qualitative analysis implied that performance of the system could differ between typical and atypical cases, which could partly explain why the performance expectancy was not higher than expected from the quantitative analysis. Participants who had ≥3 years of clinical experience stated that alerts for typical cases would most likely be ignored and could moreover cause alert fatigue. There were also concerns that the clinicians’ decisions could be biased by the system not only when the alert appears but also when it does not appear. As implied in a previous study [26], how we can prevent excessive trust in a CDSS, which can interfere with developing clinical skills in training physicians (eg, AI-based CDSSs focusing on ruling out critical conditions rather than making diagnoses), has yet to be fully explored. However, there were also positive comments that it can help improve accuracy of diagnosis and that it has the potential to decrease misdiagnosis—especially for inexperienced physicians—and reduce costs.

#### Compatibility

We found that the risk of system failure (eg, freezing of the system) is also a barrier, especially for AI-based CDSSs. In the interview, one participant reported previously experiencing system failure due to the installment of a new system, which disrupted the clinical workflow. Although some system failure issues have already been identified [27], the increasing computational resources required for AI should carefully be taken into consideration for both stand-alone CDSSs and CDSSs integrated with EHR. More importantly, providing physicians mental security that the system would not fail or freeze is crucial to user acceptance.

### Facilitators

#### Evidence Strength

User distrust in the system has also been a barrier for CDSSs, and the “black box” nature of AI-based systems are known to compound this issue [28]. Through the explanation of the data set and by visualizing the alerts in an intuitive manner, the Emergency Alert System was perceived to be a sufficient alert system. In fact, some practitioners valued the locality over the universality of the system, considering the local trends of disease. Though this can be a facilitator to implementation, the validation of the model should be performed thoroughly, as small data sets tend to overfit, leading to undesired consequences [29].

#### Design Quality

Alert fatigue is a common issue in implementing CDSSs [30], and previous studies have revealed that alert fatigue could be reduced by refining the human-machine interface and clinical role tailoring [31]. Another study suggests that poor CDSS design could worsen alert fatigue [32]. The Emergency Alert System was designed to avoid alert fatigue through real-time background processing of information and by presenting visual information. The results of the quantitative analysis were consistent with the qualitative analysis in that users’ perceived complexity of the system was low and they judged the system to be neither interruptive nor effortful. Although modifying the alert depending on the clinical role of the user (eg, nurses) is important to reducing alert fatigue, our results suggested that tailoring the system based on clinical experience would be more effective in practice.

### Potential Limitations

There are potential limitations to this study. First, the participants reported that they have limited familiarity with information technology (although they do regularly use EHRs), which may mean that the findings might not be generalizable to other physicians. However, including physicians with varying years of postgraduate training from multiple tertiary care hospitals could have at least partially addressed this limitation. Second, the results may also be affected by external settings. The data were collected in two hospitals in Japan, the majority of clinicians who participated were trainees, and the developed model and user interface are unique to the CDSS. The convenience sampling and the composition of the clinical experience of participants in this study may have been sources of bias. Nevertheless, our findings are consistent with prior studies in that the user interface and system reliability are key to user acceptance and implementation [4,33,34]. Third, the sample size was small for a quantitative analysis and has limited real-world relevance. However, given the mixed methods design of our study, the sample size for the qualitative analysis is acceptable according to earlier studies [35,36]. Lastly, the system was in the preimplementation phase. Though most participants found the simulated setting to be comparable to the actual clinical setting, the session was done online and outside of the emergency department. Thus, further studies are needed to evaluate the developed system in the clinical setting. In addition, information regarding social influence and facilitating conditions were answered on the basis of respondents’ personal knowledge and may not be consistent in the quantitative analysis.

### Conclusions

Our mixed methods evaluation based on theoretically grounded frameworks revealed the acceptance, barriers, and facilitators of implementation of AI-based CDSS. Although the concern of system failure and overtrusting of the system could be barriers to implementation, the locality of the system and designing an intuitive user interface could likely facilitate the use of optimal AI-based CDSS. Alleviating and resolving these factors should be key to achieving good user acceptance of AI-based CDSS.

## Appendix

Multimedia Appendix 1 Supplemental tables.

## Abbreviations

AI: artificial intelligence
CDSS: clinical decision support system
CFIR: Consolidated Framework for Implementation Research
COREQ: Consolidated Criteria for Reporting Qualitative Research
EHR: electronic health record
UTAUT: Unified Theory of Acceptance and Use of Technology
