# Supplementary material for: Acceptance, Barriers, and Facilitators to Implementing Artificial Intelligence–Based Decision Support Systems in Emergency Departments: Quantitative and Qualitative Evaluation
Source: JMIR Form Res. 2022 Jun 13;6(6):e36501. doi: 10.2196/36501 (PMC9237770; doi:10.2196/36501)
Supplement: Multimedia Appendix 1 [file formative_v6i6e36501_app1.docx]

**Table S1. Model cases with clinical vignette used in the session**

|  | Final Diagnosis | age | sex | Chief complaint | Past medical history | Glasgow Coma Scale | Systolic blood pressure | Diastolic blood pressure | Body temperature | Heart rate | Respiratory rate | History of present illness (summarized) |
| --- | --- | --- | --- | --- | --- | --- | --- | --- | --- | --- | --- | --- |
| 1 | Aortic Dissection*^1^ | 78 | Male | Back pain | Hypertension, chronic obstructive pulmonary disease, prostate cancer | E4V5M6 | 162 | 110 | 37.0 | 94 | 20 | Upper back suddenly started to hurt early in the morning and the pain later spread to her lower back. Loxoprofen sodium hydrate tablets did not improve pain. Slight nausea. Smokes 20 cigarettes/day. Social drinker. |
| 2 | Aortic Dissection*^1^ | 53 | Male | Chest Pain | Hypertension, dyslipidemia | E4V5M6 | 150 | 100 | 37.9 | 80 | 22 | Sudden chest pain (numerical rating scale for pain, 5 out of 10) appeared the day before presentation. The pain seemed to be gradually improving. Today, she had chest discomfort and mild nausea. She had the same symptoms about 10 years ago and last year and was under observation. Non-smoker, non-drinker. |
| 3 | Aortic Dissection*^1^ | 74 | Female | Lower back pain | Hypertension, type 2 diabetes meliticus | E4V5M6 | 200 | 130 | 36.1 | 101 | 20 | Mild lower back pain from three days before presentation. She was diagnosed as having acute back pain the day before presentation and Loxoprofen sodium hydrate tablet was given. Her pain was controlled well, but the pain relapsed. |
| 4 | Acute Cholecystitis | 53 | Male | Nausea | Hypertension, type 2 diabetes meliticus | E4V5M6 | 75 | 40 | 36.5 | 53 | 18 | Nausea and vomiting at night, the day before presentation. Last meal was a croquette burger at around 18:00. He complained of a queasy feeling in the chest, but there was no obvious chest pain. No history of nausea and chest pain. |
| 5 | Nephrolithiasis | 58 | Male | Back pain | None | E3V5M6 | 160 | 100 | 37.0 | 110 | 22 | Sudden back pain while walking with his dog. Couldn't stand up and hobbled on the spot. After that, he felt as if the pain was spreading to his upper back. Pain in his right leg had also appeared. |
| 6 | Gastritis | 69 | Female | Stomachache | Hypertension, dyslipidemia, type 2 diabetes meliticus | E4V5M6 | 130 | 65 | 36.8 | 105 | 22 | Has experienced pulsating sensation in her abdomen for years. She had been aware of abdominal pain for a week. Good appetite and no vomiting or diarrhea, tenderness in the upper umbilical region. No chest pain or back pain. |
| 7 | Myocardial infarction | 75 | Male | Chest Pain | None | E3V4M6 | 154 | 102 | 36.3 | 121 | 28 | Past smoker, 40 cigarettes/day until 10 years ago. Sudden chest and back pain while working in the fields. Lying down and resting improved the pain but resuming work made the pain worse. The pain was severe (numerical rating scale for pain, 10 out of 10), and the patient was experiencing continuous severe pain that he had never felt before. |
| 8 | Aortic Dissection*^1^ | 53 | Male | Back pain | None | E4V5M6 | 150 | 100 | 36.5 | 70 | 18 | Had pain from his back to his stomach and could not sleep. The pain did not improve until morning, and was to perceived to be different from nephrolithiasis.(the patient had a past history of nephrolithiasis) |
| 9 | Aortic Dissection*^2^ | 72 | Female | Fever | Myocardial infaction, hypertension, heart failure | E4V5M6 | 148 | 105 | 37.2 | 110 | 22 | Nausea, vomiting, and chest pain appeared three days prior to presentation. She was prescribed an antipyretic analgesic and antiemetic drugs, but her symptoms did not improve. The patient was able to drink water, but was unable to eat much. Chest pain appeared slowly in the cardiac region (e.g., near the right rib cage). Pain was exacerbated in supine position, so she was in lateral recumbent position. |
| 10 | Aortic Dissection*^1^ | 65 | Male | Back pain | None | E3V5M6 | 155 | 104 | 36.5 | 80 | 24 | He woke up with severe pain in her abdomen and back. A family member found him in pain in the middle of the night and called an ambulance. It was the first time in his life that he felt such severe pain. The patient vomited once after the emergency team arrived, but there was no hematemesis. The pain was so strong that he could not walk. The pain did not change with posture. |
| 11 | Aortic Dissection*^2^ | 53 | Male | Toothache | Aortic Dissection | E4V5M6 | 154 | 112 | 37.1 | 90 | 20 | Three days ago, there was pain in his left back teeth and left shoulder, which persisted until two days before presentation and then completely disappeared. |

Asterisk 1 (*^1^) represents typical aortic dissection

Asterisk 2 (*^2^) represents atypical aortic dissection

**Table S2. All codes and count data differentiated by Consolidated Framework for Implementation Research domain and construct**

| Constructs | Barriers | Count | Facilitators | Count |
| --- | --- | --- | --- | --- |
| I. Intervention Characteristics |  |  |  |  |
| Evidence Strength & Quality | Doubtful of the results | 1 | sample size was enough for developing models | 9 |
|  |  |  | local trends of disease | 1 |
| Relative Advantage | Unnecessary for experienced emergency physicians | 1 | Potential to reduce misdiagnosis | 1 |
|  | Unnecessary for typical cases | 6 | Useful than diagnostic rules | 2 |
|  | alternatives for the system is enough | 2 | Never seen similar systems | 8 |
|  | Can bias physician's decision | 2 | Can aid diagnosis for difficult cases | 6 |
|  | Limited use cases | 1 | Good for information sharing | 1 |
|  |  |  | Useful for unexperienced physicians | 3 |
| Design Quality & Packaging | Unable to find when the system showed alerts | 1 | Easy to use and not interruptive | 10 |
|  | Potentially distracting for comorbidities | 1 | Summary board was informative | 1 |
|  |  |  | Real-time alerts were intuitive | 2 |
| Adaptability | Long loading time | 1 | Alert of many diseases are helpful | 1 |
|  | Alert of many disease can be complex | 1 |  |  |
| III. Inner Settings |  |  |  |  |
| Culture | The hospital is not tolerant of changes | 1 |  |  |
| Tension for Change |  |  | Have experience of nearly misdiagnosing | 2 |
|  |  |  | Thought of using alert systems | 2 |
| Compatibility | Anxious if the system is working properly | 1 | Easily integrated with existing workflow | 14 |
|  | Affects typing speed | 1 |  |  |
|  | Fear to system failure | 1 |  |  |
| Culture | The hospital is not tolerant of changes | 1 |  |  |
| Tension for Change |  |  | Have experience of nearly misdiagnosing | 2 |
|  |  |  | Thought of using alert systems | 2 |
| Relative Priority | Assessing and stabilizing the patient should be prioritized to writing the medical record | 2 |  |  |
| Access to Knowledge & Information | Cannot understand how the system works | 1 | The demonstration was sufficient for usage |  |
|  | Not familiar with computer systems | 1 |  | 2 |
| IV. CHARACTERISTICS OF INDIVIDUALS |  |  |  | 2 |
| Knowledge & Beliefs about the Intervention | Unnecessary for educational facilities | 1 | I would like to use it | 8 |
|  |  |  | I would like to use it if the system is more developed | 1 |
